# Down-regulation of MAPK pathway alleviates TRPV4-mediated trigeminal neuralgia by inhibiting the activation of histone acetylation

**DOI:** 10.1007/s00221-021-06194-6

**Published:** 2021-09-09

**Authors:** Weidong Liu, Benfang Pu, Mindi Liu, Xuejun Zhang, Ran Zeng

**Affiliations:** 1 Department of Neurosurgery, Shanghai Punan Hospital of Pudong New District, 279 Linyi Road, Pudong New District, Shanghai, 200125 China; 2 The Second Department of Neurosurgery, Shanghai Donglei Brain Hospital, 988 Huaxu highway, Qinpu district, Shanghai, 201702 China

**Keywords:** MAPK, TRPV4, Trigeminal neuralgia, Histone acetylation

## Abstract

Our objective of this study is to determine the molecular mechanism of MAPKs (mitogen activated protein kinase systems) on TRPV4 (transient receptor potential vanilloid 4)-mediated trigeminal neuralgia (TN). Partial chronic constriction injury of the infraorbital nerve (CCI-ION) ligation model was used in this research. When treated with antagonists of p38, JNK or ERK, the mechanical hyperalgesia threshold, nerve fiber disorder, myelinoclasis, and Schwann cells proliferation could be reversed. RT-PCR (real-time quantitative polymerase chain reaction), Western blot and IHC (immunohistochemistry) showed that TRPV4 mRNA and protein levels, TRPV4-positive cells and small positive neurons decreased remarkably in TN group treated with antagonists of p38, JNK or ERK. ELISA (enzyme-linked immunosorbent assay) was performed to discover inhibition of MAPK pathway can down-regulate the expression of HATs (histone acetyltransferases), and up-regulate the expression of HDACs (histone deacetylases) in TN, thus inhibiting histone acetylation. Finally, Western blot was performed to identify the phosphorylation status of p38, JNK and ERK, finding decreased phosphorylation forms in antagonists treated TN groups compared with TN groups. Based on the above investigation method, on a whole, our study showed that down-regulation of MAPK pathway could alleviate TRPV4-mediated trigeminal neuralgia, via inhibiting the activation of histone acetylation.

**Supplementary Information:**

The online version contains supplementary material available at 10.1007/s00221-021-06194-6.

## Introduction

Trigeminal neuralgia (TN) is considered as one of the most painful and prevalent neuropathic disorders of repeated episodes. It is known as stabbing pains or paroxysmal attacks of one-sided intense electric shock-like in areas supplied by the trigeminal nerve. The incidence rate of TN is increasing as ages (Katusic et al. 1990; Mueller et al. 2011). TN is associated with trigeminal nerve injury, leading to chronic pain such as burning and aching (Eide and Rabben 1998; Fried et al. 2001). Oral administration is preferred for trigeminal neuralgia treatment. Surgery could be optional when the oral administration does not work. However, the results are usually not satisfied after patients have undergone a variety of treatments.

There are a great variety of ion channels in TNs. Family of transient receptor potential (TRP) is an important ion channel family discovered lately. The basic properties and function of TRPVs (Transient receptor potential vanilloid receptors) suggests some members of this subfamily are important for perception of pain, temperature sensitivity, osmotic regulation and maintenance of Ca^2+^ homeostasis (Lappin et al. 2006; Costa et al. 2010). Based on structure and function, mammalian TRPVs are classified into four groups: TRPV-1/TRPV-2, TRPV-3, TRPV-4, and TRPV-5/TRPV-6. In addition, TRPV4 has received increasing attentions these years in trigeminal neuropathic pain (Urano et al. 2012). Previous studies indicated that TRPV4 was one of the main causes of TNs, allodynia and hyperalgesia (Shibasaki et al. 2007). TRPV4 siRNA interference could induce partial reversal of pain threshold decreasing. However, the pathways and mechanism of TRPV4 inducing pains still need further study.

Peripheral mitogen activated protein kinase systems (MAPKs) are widely involved in the nervous system, mainly leading to three subsystems, extracellular signal regulated kinases (ERK), c-Jun N-terminal kinase (JNK) and hypertonic glycerol kinase (p38) which are the main effected factors activated as phosphorylated forms (Ji et al. 2009). MAPK signal pathways play key roles in extracellular stimuli changing to intracellular transcription and translation reaction (Cao et al. 2013). Studies confirmed that the initiation and development of neuropathic pains is closely associated with MAPK signaling pathway (Obata and Noguchi 2004).

Histone acetylation plays critical roles in post-transcription regulating progression of histone leading to accumulation of protein/transcription factor complex. The regulation of histone acetylation is a reversible dynamic procedure relying on the activities of HATs (histone acetyltransferases) and HDACs (histone deacetylases) (Gurard-Levin and Almouzni 2014; Ruiz-Garcia et al. 1998). It can regulate gene transcription by changing the structure conformation of chromatin. It is reported that the increasing acetylation of histone H3 resulted in the up-regulation and transcription of pain-related genes. And previous study found that MAPK pathways could activate epigenetic histone acetylation.

Thus, we speculated that TRPV4 participated in the central and peripheral sensitization mechanism of neuropathic pain, and TRPV4 channel was regulated by the expression and activity of MAPK pathway related to histone acetylation.

Based on the above progresses, in this study, we detected the function of TRPV4***-MAPK pathway in neuropathic pain and explored a new theory of trigeminal neuralgia.

## Materials and methods

### Experimental animals

Adult male Sprague–Dawley (SD) rats, weighing 200–300 g, were randomly assigned to seven groups, which were bought from Shanghai SLAC Laboratory Animal Co. The animals were raised at 23 °C, light alternant of 12 h white/12 h dark. All procedures for animal care were approved by the Animal Management Committee of Shanghai Punan Hospital of Pudong New District. All animal experiments were performed in compliance with the Guidelines for Proper Conduct of Animal Experiments established by the Animal Management Committee.

### Partial chronic constriction injury of the infraorbital nerve (CCI-ION) ligation model

In brief, the TN group (TN) was treated by ION partially ligated using silk according to Seltzer’s model (Seltzer et al. 1990). The rats of control group (Control) were raised without any treatment. For the sham operation control group (Sham), a sham operation was performed identically except ligating nerve. The normal saline group (TN + NS) was injected with normal saline on an empty stomach four hours before TN model form. The p38 inhibitor group (TN + p38) was injected with p38 inhibitor SB203580 (CST, Beverly, USA) on an empty stomach 4 h before TN model form. The JNK inhibitor group (TN + JNK) was injected with JNK inhibitor SP600125 (CST, Beverly, USA) on an empty stomach four hours before TN model form. The ERK inhibitor group (TN + ERK) was injected with ERK inhibitor U0126 (CST, Beverly, USA) on an empty stomach four hours before TN model form.

### Mechanical hyperalgesia threshold analysis

The mechanical hyperalgesia threshold was measured by the von Frey filaments (Nakatsuka and Iwai 2009) 1 day before surgical operation and 3, 5, 7, 14 days after surgical operation. The intensity of the strength is variational from 5 to 35 g on rats’ facial area around nose. The stimuli were repeated six times and mean data were acquired. When rats reacted like going backwards, attacking, or scratching operational side face constantly, the intensity used would be the mechanical hyperalgesia threshold.

### HE staining (hematoxylin and eosin staining)

The slices of trigeminal ganglions (TG) were stained with hematoxylin and eosin after pre-processed with xylol and ethyl alcohol of different concentrations. After being rinsed with ethyl alcohol for three times and xylol for one time, tissues were immobilized with wood gum and dried later. Thereafter sections were detected by Image-Pro Plus software.

### Real-time quantitative polymerase chain reaction (RT-qPCR)

The mRNA expression levels of TRPV4 were measured by RT-PCR. Total RNA was extracted from TGs respectively and reversely transcribed to cDNA with a first strand cDNA kit (cat no, 11117831001, Sigma, Munich, Germany), according to the protocol provided by the manufacturer. PCR amplification was performed for 30 s at 95 °C, followed by 40 cycles: denaturation at 95 °C for 5 s, annealing/extension at 60 °C for 30 s in ABI 7300 Thermocycler (Applied Biosystems, Foster City, CA, USA) using the SYBR Premix Ex Taq kit (Takara, Dalian, China). The primer sequences of TRPV4 were 5′ CCCTTGCTCCCACCTACATT 3′ and 5′ TCCATGGGAGTGAGACAGGT 3′ (products: 195 bp).

### Western blot analysis

The concentrations of proteins were determined by BCA assay (Beyotime, Nantong, China). Then proteins were subjected to sodium dodecyl sulfate-polyacrylamide gel electrophoresis (SDS-PAGE) and electroblotted onto a polyvinylidene fluoride (PVDF) membrane (Amersham, UK). Following blockage with 5% nonfat dry milk in PBS for 1 h, the blotting membranes were probed overnight, respectively, at 4 °C, with the TRPV4 primary antibodies, then they were probed with the appropriate HRP-conjugated secondary antibodies. The PVDF membrane was exposed to X-ray film and immunoreactive bands were detected by reaction with enhanced chemiluminescence (ECL) detection system reagents (Amersham, Arlington Heights, IL, USA). For loading control, the membrane was probed with a monoclonal antibody for glyceraldehyde-3-phosphate dehydrogenase (GAPDH). Lab Works Image Acquisition and Analysis Software (UVP, Upland, CA, USA) was used to quantify band intensities. Antibodies were purchased from Abcam (Cambridge, UK).

### Immunohistochemistry (IHC)

Trigeminal ganglions (TG) were fixed and processed for immunohistochemistry. Animals were immobilized with cold 4% paraformaldehyde (PFA). After being rinsed for three times, sections were reacted with primary antibody overnight at 4 °C. Then secondary antibodies were applied to the sections for 30 min. Thereafter, the sections reacted with coloring agent DAB (diaminobenzidine), cover-slipped and detected by Image-Pro Plus software. No specific labeling was observed in the absence of a primary antibody. Antibodies were purchased from Abcam (Cambridge, UK).

### ELISA (enzyme-linked immunosorbent assay)

The quantities of HATs and HDACs were determined by ELISA kit (R&D, USA), following the manufacturer’s instructions. Samples and standard substances were added into wells in 96-well plate. After incubation for 90 min at 37 °C biotinylated antibodies were added into the wells and incubated for 60 min at 37 °C. After being rinsed for 3 times, ABC (avidin peroxidase complex) were added in and incubated for another 30 min. After being rinsed for 3 times, TMB (tetramethylbenzidine) coloring regents were added in and incubated for 15 min at 37 °C. Finally, the optical density (OD) values were read at 450 nm by a microplate reader (Thermo, USA). The sample quantities could be calculated by standard curve.

### Phosphorylation detection

The phosphorylation levels of factors (p38, JNK and ERK) in MAPK pathway were measured by Western blot described above.

### Statistical analysis

All results were expressed as mean ± standard deviations of three independent experiments. Statistical analysis was performed using a SPSS 13.0 statistical package and data were subjected to one-way analysis of variance (ANOVA), followed by Dunnett’s test. *p* < 0.05 was considered significant; *p* < 0.01 was considered especially significant.

## Results

### Function of antagonists of p38, JNK and ERK on CCI-ION-induced physiological variation

**Fig. 1:**
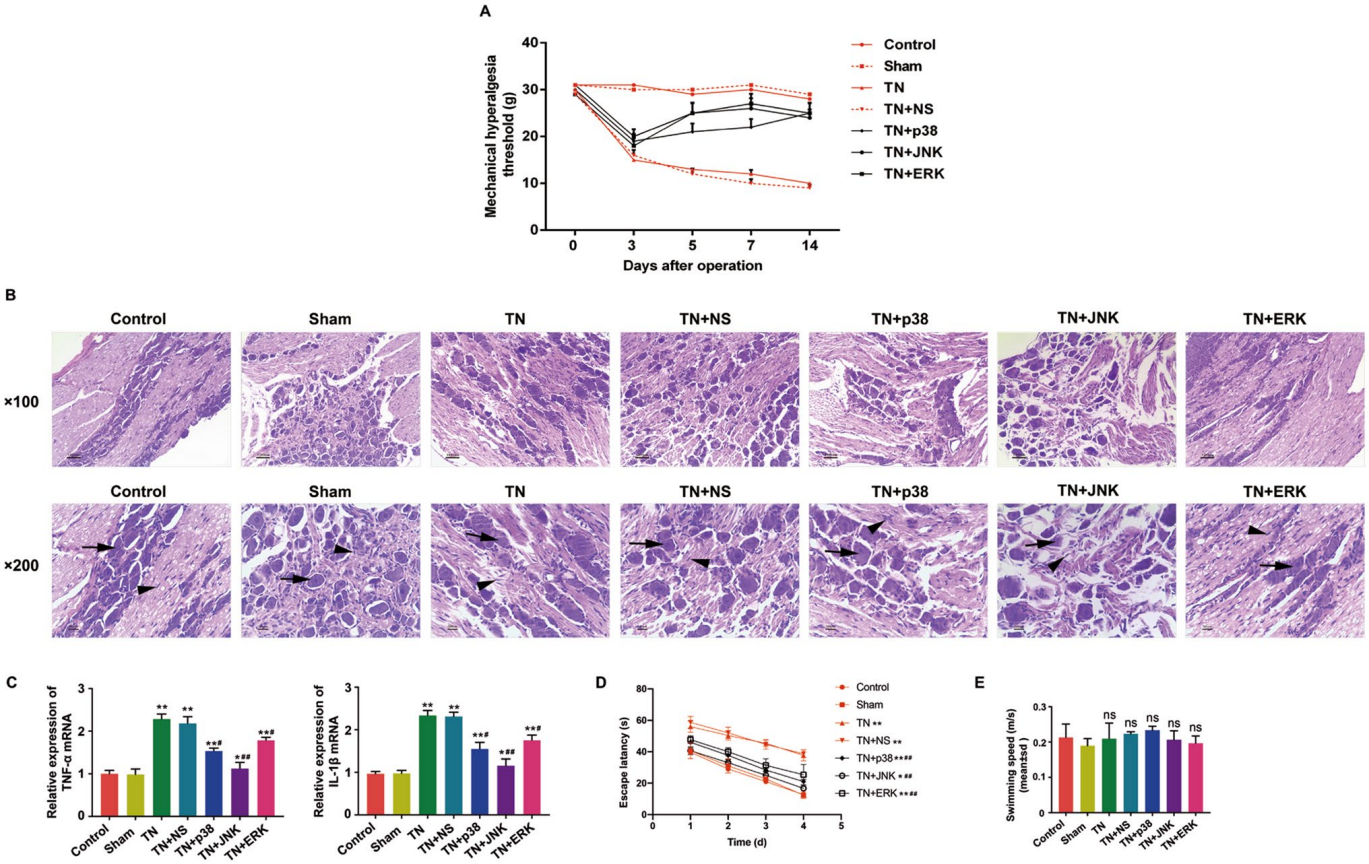
Function of antagonists of p38, JNK and ERK on CCI-ION-induced physiological variation. **A** The mechanical hyperalgesia thresholds were measured by the von Frey filaments (Nakatsuka and Iwai 2009) 1 day before and 3, 5, 7, 14 days after surgical operation. **B** The histological changes were measured by HE staining (scale plate: 100 µm and 200 µm). **C** The expression levels of TNF-α and IL-1β were detected by RT-qPCR. **D** Escape latency in reaching the platform of different groups in the experiments of Morris Water Maze. **E** Swimming speed between all groups in the experiments of Morris Water Maze. Data were presented as mean ± SD, n = 10 per group, *p < 0.05 vs. sham control, **p < 0.01 vs. sham control, ^#^*p* < 0.05 vs. TN group, ^##^*p* < 0.01 vs. TN group

The mechanical hyperalgesia thresholds were measured by the von Frey filaments (Nakatsuka and Iwai 2009) 1 day before and 3, 5, 7, 14 days after CCI-ION-related surgical operations. The control and sham control's mechanical hyperalgesia thresholds stayed invariable levels during the whole process. The TN and TN + NS groups declined from 3 to 14 days after the operation. Otherwise, when treated with antagonists of p38, JNK and ERK (named SB203580, SP600125, U0126), respectively, before operation, the mechanical hyperalgesia threshold first declined in 3 days similar to TN group, then rose up till 14 days after operation, when the mechanical hyperalgesia thresholds nearly recovered to the data of control group (Fig. 1A).

The histological changes were measured by HE staining. As is shown in Figs. 1B and S1, the nerve fibers in control and sham control tissues were equally distributed. On the other hand, nerve fibers were arranged in disorders, with damaged myelinoclasis and inflammatory cell infiltration in TN and TN + NS groups compared with control and sham control. As expected, less myelinoclasis damage and inflammatory cell infiltration was observed in TN + p38, TN + JNK and TN + ERK groups.

It suggested that down-regulation of MAPK pathway could increase the mechanical hyperalgesia thresholds of TN and repair the disorders of the nerve fibers, myelinoclasis and so on. To further validate our findings, we detected mRNA levels of proinflammatory cytokines including TNF-α and IL-1β in groups. Consistent with the results of HE staining, the expression levels of TNF-α and IL-1β in TN + p38, TN + JNK and TN + ERK groups were significantly lower than that in TN and TN + NS groups (Fig. 1C).

We also conducted the experiments of Morris Water Maze to evaluate the cognitive performance of rats in groups. The results showed that rats in TN and TN + NS groups showed significantly longer escape latency in reaching the platform than the control and sham group, indicating that trigeminal neuralgia resulted in cognitive impairment (Fig. 1D). On the contrary, rats injected with antagonists of p38, JNK and ERK presented a significant decrease in escape latency. However, no significant differences were observed in swimming speed between all groups (Fig. 1E).

### Function of antagonists of p38, JNK and ERK on CCI-ION-induced TRPV4 mRNA and protein expression levels and TRPV4-positive neuron variation

**Fig. 2:**
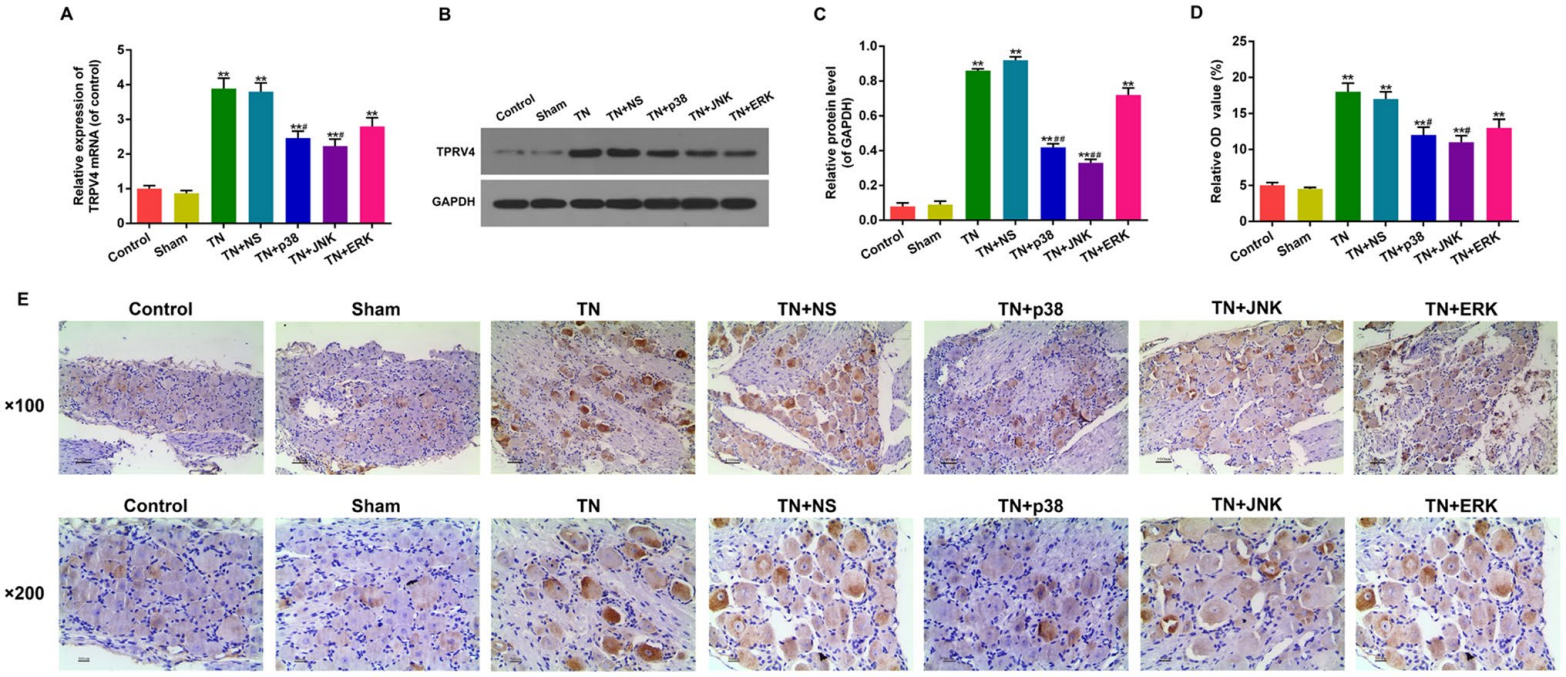
Function of antagonists of p38, JNK and ERK on CCI-ION-induced TRPV4 mRNA and protein expression levels and TRPV4-positive neuron variation. **A** The mRNA expression levels of TRPV4 were measured by RT-PCR. **B**, **C** Protein levels of TRPV4 were measured by Western blot. GAPDH was detected as the control of sample loading. **D**, **E** TRPV4-positive nerve cell distribution was measured by immunohistochemistry (scale pate: 100 µm and 200 µm). Data were presented as mean ± SD, *n* = 10 per group, *p < 0.05 vs. sham control, **p < 0.01 vs. sham control, ^#^*p* < 0.05 vs. TN group, ^##^*p* < 0.01 vs. TN group

The mRNA and protein expression levels of TRPV4 were measured by RT-PCR and Western blot. As shown in Fig. 2A, B and C, mRNA and protein levels of TRPV4 increased notably in TN and TN + NS groups compared with control and sham control. Meanwhile, mRNA and protein levels of TRPV4 in TN + p38, TN + JNK and TN + ERK groups treated with antagonists of p38, JNK and ERK (named SB203580, SP600125, U0126), respectively, before operation decreased compared with TN and TN + NS groups, but still higher than control and sham control. But the expression of TRPV1 changed marginally (Figure S2). As shown in Fig. 2D and E, the results of immunohistochemistry showed that TRPV4-positive cells and small positive neurons increased remarkably in TN and TN + NS groups compared with control and sham control. TRPV4-positive cells and small positive neurons of TN + p38, TN + JNK and TN + ERK groups treated with antagonists before the operation decreased compared with TN and TN + NS groups, but still higher than control and sham control. Notably, the combination of three antagonists showed comparable effect on the expression of TRPV4 (Figure S3A).

It suggested that down-regulation of MAPK pathway could inhibit TRPV4 expression critical in the onset of TN.

### Function of antagonists of p38, JNK and ERK on CCI-ION-induced histone acetylation

**Fig. 3:**
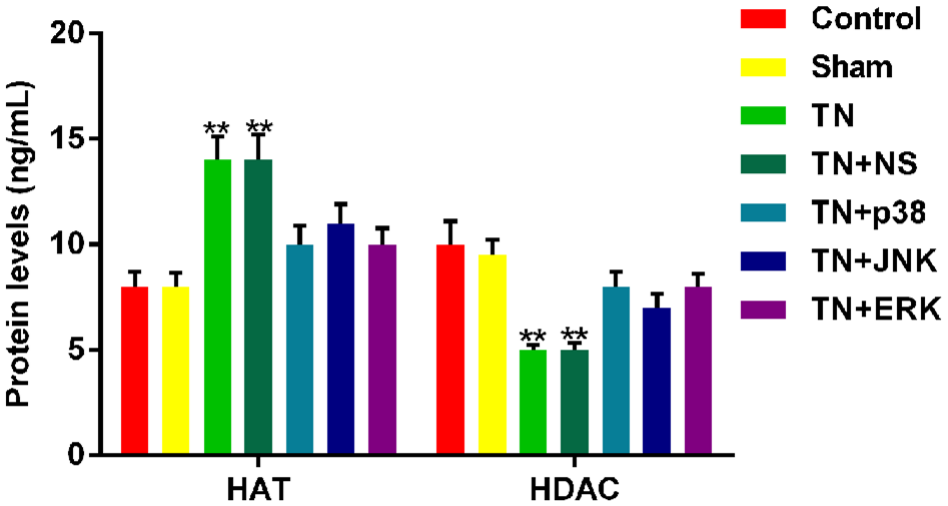
Function of antagonists of p38, JNK and ERK on CCI-ION-induced histone acetylation. The protein levels of HATs and HDACs were measured by ELISA. Data were presented as mean ± SD, *n* = 10 per group, *p < 0.05 vs. sham. control, **p < 0.01 vs. sham control, ^#^*p* < 0.05 vs. TN group, ^##^*p* < 0.01 vs. TN group

HATs and HDACs are important factors in histone acetylation. HATs can promote histone acetylation, while HDACs reverse the histone acetylation. ELISA was performed to determine the protein levels. As shown in Fig. 3, protein levels of HATs increased notably in TN and TN + NS groups compared with control and sham control. Protein levels of HATs in TN + p38, TN + JNK and TN + ERK groups treated with antagonists before the operation decreased compared with TN and TN + NS groups, but still higher than control and sham control. Otherwise, HDAC protein levels decreased remarkably in TN and TN + NS groups compared with control and sham control. HDAC protein levels in TN + p38, TN + JNK and TN + ERK groups increased compared with TN and TN + NS groups, but still higher than control and sham control. Interestingly, the combination of three antagonists and HDAC inhibitor showed reverse effect on the expression of TRPV4 (Figure S3B).

### Function of antagonists of p38, JNK and ERK on CCI-ION-induced phosphorylation in MAPK pathway

**Fig. 4:**
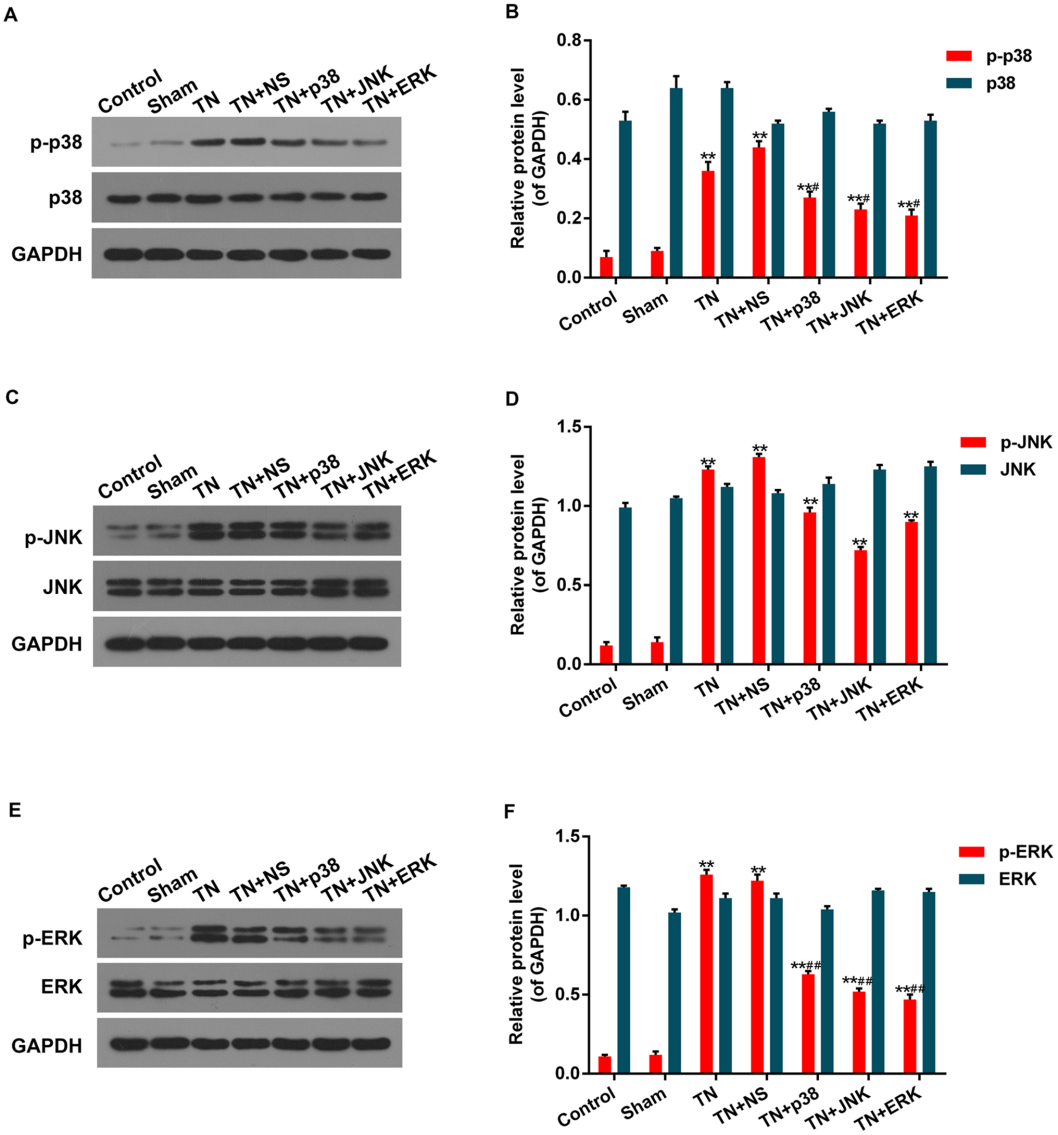
Function of antagonists of p38, JNK and ERK on CCI-ION-induced phosphorylation in MAPK pathway. **A** Protein levels of p-p38 and p38 were determined by Western blot. **B** Protein levels of p-JNK and JNK were determined by Western blot. **C** Protein levels of p-ERK and ERK were determined by Western blot. GAPDH was detected as the control of sample loading. Data were presented as mean ± SD, *n* = 10 per group, *p < 0.05 vs. sham control, **p < 0.01 vs. sham control, ^#^*p* < 0.05 vs. TN group, ^##^*p* < 0.01 vs. TN group

The phosphorylation levels of factors (p38, JNK and ERK) important in MAPK pathway increased notably in TN and TN + NS groups compared with control and sham control, While the phosphorylation levels of them in TN + p38, TN + JNK and TN + ERK groups treated with antagonists before the operation decreased compared with TN and TN + NS groups, but still higher than control and sham control (Fig. 4). Meanwhile different groups had similar non-phosphorylation levels of these proteins.

## Discussion

Trigeminal neuralgia is constant and severe orofacial pain syndromes which is caused by nerve compression and characterized by sudden onset of stabbing pains (Eide and Rabben 1998; Fried et al. 2001; Turp and Gobetti 1996). Members of TRPVs such as TRPV4 are considered as one of the main cause of TNs, allodynia and hyperalgesia. MAPK pathways, widely existing in the nervous system, play critical roles in extracellular stimuli changing to intracellular transcription and translation reaction. But the mechanism of MAPK pathway on trigeminal neuralgia still needs further research.

The primary objective of this study is to elucidate the major molecular mechanism of MAPK pathway on TRPV4-mediated trigeminal neuralgia. Partial chronic constriction injury of the infraorbital nerve (CCI-ION) ligation model was used in this research. A previous study by Hirono et al. and our study are partially inspired by this study. Hirono et al. has demonstrated that TRPV1 plays a crucial role in trigeminal neuropathic pain. However, the upstream regulatory mechanism of TRPV1 remains largely unknown. In this study, we investigated the regulatory role of MAPK signaling pathway on the expression level of TRPV1 and TRPV4. We found that down-regulation of MAPK pathway could alleviate TRPV4-mediated trigeminal neuralgia, via inhibiting the activation of histone acetylation. But the expression of TRPV1 was marginally changed with the modulation of MAPK signaling pathway (Figure S2). The initial observation showed that mechanical hyperalgesia threshold declined in TN, with nerve fibers in disorders, myelinoclasis, Schwann cell proliferation and so on. When treated with antagonists of p38, JNK or ERK, the mechanical hyperalgesia threshold and the phenomenon of nerve fibers in disorders, myelinoclasis, and Schwann cell proliferation could be reversed. It indicated that p38, JNK and ERK in MAPK pathway playing critical roles in promoting the onset of TN. Down-regulation of MAPK pathway could increase the mechanical hyperalgesia thresholds of TN and recover the disorders of the nerve fibers, myelinoclasis and so on, consequently inducing partially alleviation of TN.

TRPV4 is Ca^2+^ high-permeability transmembrane ion channel, one of the main causes of TNs, allodynia and hyperalgesia (Clapham 2003). Our study showed that TRPV4 mRNA and protein levels, TRPV4-positive cells and small positive neurons increased remarkably in TN group compared to the control group. When treated with antagonists of p38, JNK or ERK, all of them were reversed notably, though still higher than control. For TRPV4 has been reported to induce TN, the results indicated that down-regulation of MAPK pathway could inhibit the expression of TRPV4, thereby decrease the pains of TN. And small neurons maybe the main neurons function in TN, to transmit achy signal. This tempted us to conduct further experiments to reveal the molecular mechanical relationship between MAPK pathway and TRPV4-mediated TN.

Histone acetylation can be promoted by HATs (histone acetyltransferases) and inhibited by HDACs (histone deacetylase), regulating the transcription of pain-related genes (Slevogt et al. 2006). And histone acetylation can also be activated by MAPK pathways (Correa et al. 2011; Schmeck et al. 2005). Therefore, we performed ELISA to detect the levels of HATs and HDACs in TN group and MAPK antagonists treated TN groups, finding that the inhibition of MAPK pathway can surely down-regulate the expression of HATs, and up-regulate the expression of HDACs. Thus, down-regulation of MAPK pathway may alleviate TRPV4-mediated TN via inhibiting the activation of histone acetylation.

The initiation and development of neuropathic pains is closely associated with MAPK signaling pathways. p38, JNK and ERK are the three important subfamilies of MAPK. ERK is a kind of Ser/Thr protein kinase, which can be activated by many stimulating factors such as growth factors, cancer genes and so on, and regulates the activity of transcriptional factors. JNK can activate the transcriptional activity of AP1 (activating protein) by phosphorylating c-jun. There are three subtypes of JNK, among which JNK3 is mainly expressed in nerve cells and regulate apoptosis of nerve cells (Davis 2000; Zhong et al. 2016). p38 is the third discovered MAPK in mammals. p38 can be activated by intracellular inflammatory factors (such as IL-1 and TNF-α) and other cell stimulating factors (such as UV, heat shock and so on), activating intracellular signal cascades and participating in cell achy and stress response. The common point of them is the phosphorylation function forms. The more phosphorylation forms exist, the greater activating function they play. So, we detected the phosphorylation degree of p38, JNK and ERK in TN and antagonists treated TN groups to investigate the relationship between phosphorylation and TN. By performing Western blot, we discovered that the phosphorylation levels of p38, JNK and ERK increased notably in TN group compared with control. When treated with antagonists, the phosphorylation levels could be decreased, respectively. It indicated that MAPK pathway played important roles in TRPV4-mediated TN through the phosphorylation of p38, JNK and ERK.

## Conclusions

In conclusion, our study showed that down-regulation of MAPK pathway could alleviate TRPV4-mediated trigeminal neuralgia, via reducing the phosphorylation degree of p38, JNK or ERK, and inhibiting the activation of histone acetylation, finally it could reach the goal of recovering normal nerve histological forms and relieving pains of TN. It may provide novel effective therapeutic strategy for trigeminal neuralgia.

## Supplementary Information

Below is the link to the electronic supplementary material.

Supplementary file1 (DOCX 8431 KB)

## Data Availability

Data are available upon request.
