# Supplementary material for: Down-regulation of MAPK pathway alleviates TRPV4-mediated trigeminal neuralgia by inhibiting the activation of histone acetylation
Source: Exp Brain Res. 2021 Sep 9;239(11):3397–404. doi: 10.1007/s00221-021-06194-6 (PMC8541954; doi:10.1007/s00221-021-06194-6)

**Supplements**

Figure legends

Figure S1 The histological changes and expression of neuronal marker map2 were measured by immunohistochemistry (scale bar: 25 µm).

Figure S2 The expression levels of TRPV1 were measured by Western blotting in all groups. All the experiments were conducted in three times.

Figure S3 The expression levels of TRPV4 were measured by RT-PCR in corresponding treatments. (A) The expression levels of TRPV4 were measured by RT-PCR in MAPK inhibitor alone or the combination of three inhibitors. (B) The expression levels of TRPV4 were measured by RT-PCR in MAPK inhibitor alone or the combination of three inhibitors and HDAC inhibitor.

Figure S1


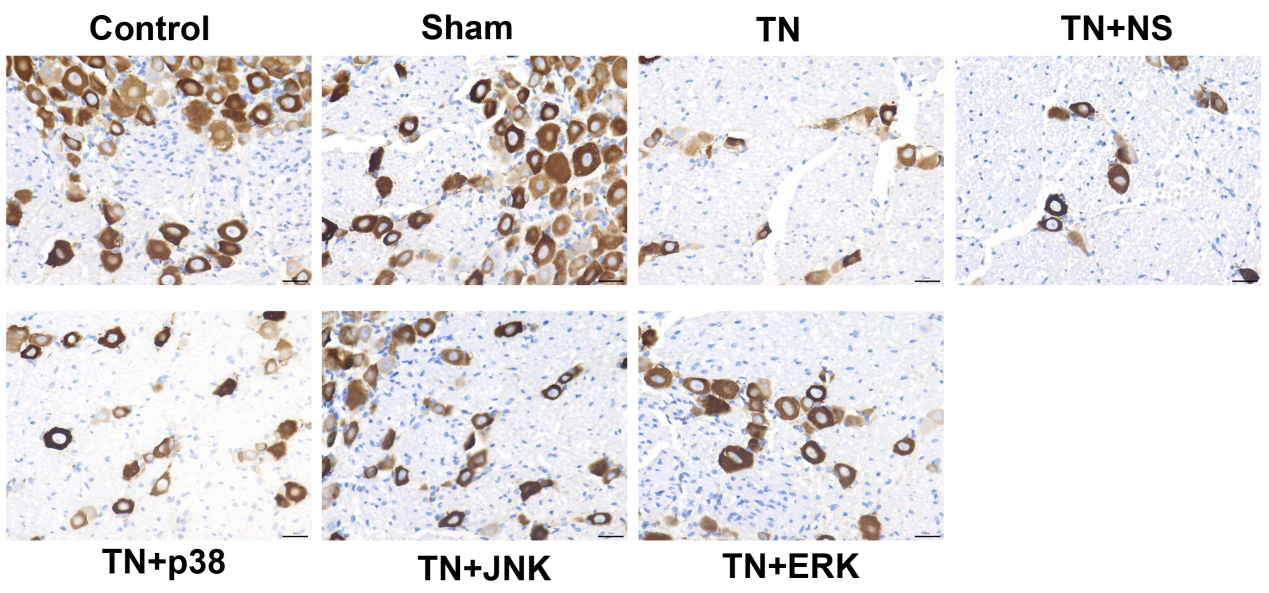


Figure S2


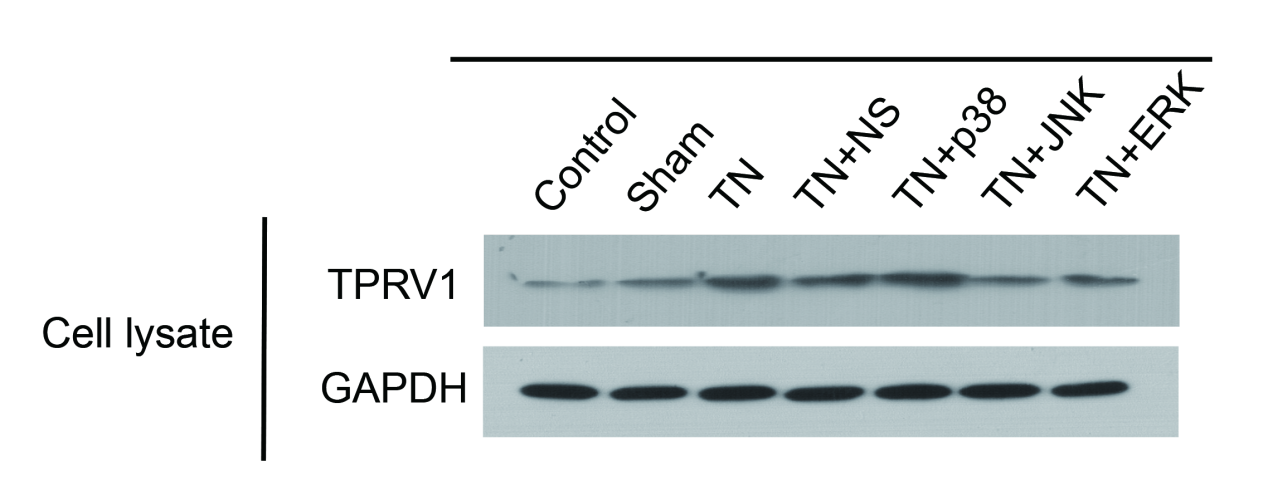


Figure S3


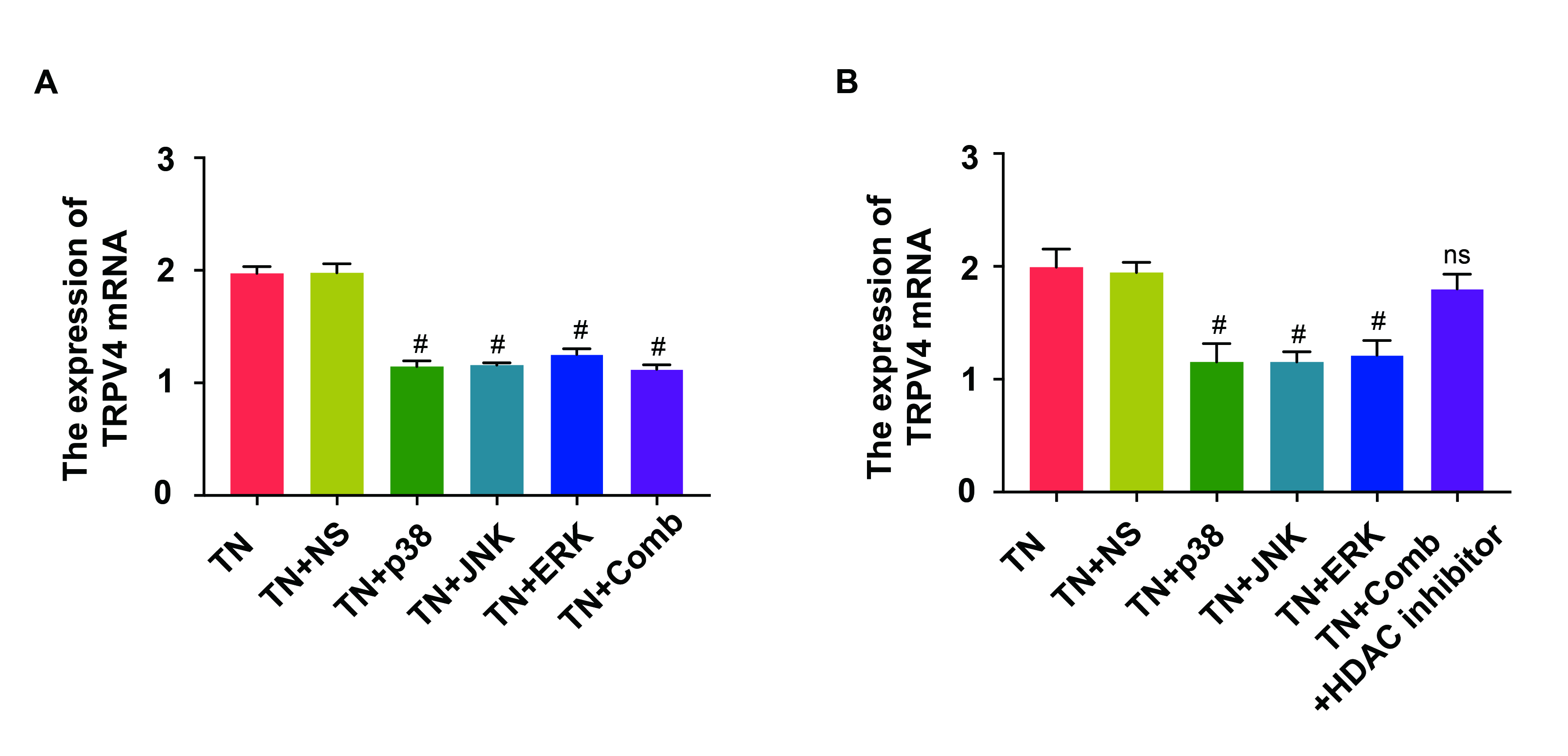

Supplement: Supplementary file 1 — Supplementary file1 (DOCX 8431 KB) [file 221_2021_6194_MOESM1_ESM.docx]
